# A Strength Training Program for Primary Care Patients, Central Pennsylvania, 2012

**DOI:** 10.5888/pcd11.130403

**Published:** 2014-06-26

**Authors:** Christopher N. Sciamanna, Vijay A. Patel, Jennifer L. Kraschnewski, Liza S. Rovniak, Dino A. Messina, Heather L. Stuckey, William J. Curry, Cynthia H. Chuang, Lisa L. Sherwood, Stacy L. Hess

**Affiliations:** Author Affiliations: Vijay A. Patel, Jennifer L. Kraschnewski, Liza S. Rovniak, Heather L. Stuckey, William J. Curry, Cynthia H. Chuang, Lisa L. Sherwood, Stacy L. Hess, Penn State Hershey Medical Center, Hershey, Pennsylvania; Dino A. Messina, Danbury Hospital, Danbury, Connecticut.

## Abstract

**Introduction:**

Primary care providers can recommend strength training programs to use “Exercise as Medicine,” yet few studies have examined the interest of primary care patients in these programs.

**Methods:**

We conducted a cross-sectional survey of primary care patients in central Pennsylvania. Interest in participating in free group-based strength training and weight control programs was assessed, in addition to patient demographics, medical history, and quality of life.

**Results:**

Among 414 patients, most (61.0%) were aged 54 or older, and 64.0% were female. More patients were interested in a strength training program (55.3%) than in a weight control program (45.4%). Nearly three-quarters (72.8%) of those reporting 10 or more days of poor physical health were interested in a strength training program compared with 49.5% of those reporting no days of poor physical health. After adjusting for potential confounders, those reporting poorer physical health had 2.7 greater odds (95% confidence interval, 1.4–5.1) of being interested in a strength training program compared with those reporting better physical health. Patients with hypertension, diabetes, or high cholesterol were not more interested in a strength training program than those without these conditions.

**Conclusion:**

Primary care practices may consider offering or referring patients to community-based strength training programs. This study observed high levels of interest in these widely available programs. Practices may also consider screening and referring those with poorer physical health, as they may be the most interested and have the most to gain from participating.

## Introduction

Strength training (ST) is one of the most powerful interventions to improve the health of older adults. Clinical trials by Nelson and colleagues found that ST improves strength in seniors by 113% in 12 weeks (1), and Fiatarone and colleagues found that ST led to large gains in muscle mass and bone density after 12 months (2). More recently, a study by Candow and colleagues found that after only 6 months of ST, men in their 60s regained enough muscle strength to resemble men in their 20s (3). ST programs also have unusually high rates of adherence. Compared with weight control programs, which retain only 10% of participants after 1 year, ST programs have nearly 50% of individuals still participating over the same time period (4,5).

ST and aerobic exercise differ in that ST requires fewer repetitions at a higher resistance, with brief rest periods between exercises (6). In contrast, aerobic exercise (ie, brisk walking or running) typically consists of continuous activity at a lower resistance. ST exercises are designed to lead to large increases in muscle size and strength, with much smaller gains in aerobic fitness. The 2011 Position Stand from the American College of Sports Medicine (ACSM) suggests that the resistance of each repetition in ST should be in the “moderate” to “hard” perceived effort range, defined as 60% to 70% of the maximum load that can be moved in a single repetition (7). This typically allows a user to complete 8 to 12 repetitions per set, which should produce muscle fatigue but not exhaustion. A rest period, typically 1 to 3 minutes, is recommended between sets of repetitions (7). The ACSM and American Heart Association (AHA) joint guidelines for adults recommend ST activities for a minimum of 20 minutes two or more days each week (6).

Despite the rapid improvements from ST and the high rates of adherence, less than one-quarter of adults meet the ACSM/AHA guidelines for ST (8). In an attempt to increase physical activity in the United States, the ACSM and American Medical Association (AMA) launched the “Exercise is Medicine” initiative in 2007, encouraging health care providers to recommend physical activity as part of routine medical care (9). Several community-based ST programs have been created and disseminated, including StrongWomen (10), Enhance Fitness (11), and Silver Sneakers. Insurance plans have also identified the benefits of ST; Silver Sneakers is included as an insurance benefit for more than 7.8 million older US adults and is available at more than 10,000 fitness centers in the United States (12,13).

What remains unknown is whether primary care patients are interested in participating in ST programs and, if so, which patients are most interested. The objective of this study was to understand how to apply the ACSM/AMA recommendations in a primary care practice.

## Methods

We designed a questionnaire to assess whether primary care patients are interested in ST programs. Because programs such as Silver Sneakers (www.SilverSneakers.com) are provided at no cost to patients, our questionnaire also presumed that the programs would have no cost. We created 2 survey items, 1 to measure interest in a free ST program and a second, a comparator, to measure interest in a free weight control program (4): “Would you consider participating in any of the following programs, if they were provided FREE to you: (A) a group weight control program led by someone who had been successful at losing weight, (B) a group program to increase your muscle strength.” Responses were “yes” or “no.” The questionnaire items were designed to have face validity, for quality improvement purposes, and were not formally tested for validity or reliability.

Mental and physical quality of life were assessed using 2 questions from the Healthy Days Measure from the Centers for Disease Control and Prevention (CDC): “Now thinking about your physical health, which includes physical illness and injury, for how many days during the past 30 days was your physical health not good?”; “Now thinking about your mental health, which includes stress, depression, and problems with emotions, for how many days during the past 30 days was your mental health not good?” (14). Self-reported health was assessed by using a single question that has been shown to predict future hospitalization and death (“Would you say that in general your health is excellent, very good, good, fair, or poor?”) (15). Physical activity was assessed by using a single-item measure developed by Greenwood and colleagues (“How many days during a typical week do you perform physical activities where your heart beats faster and your breathing is harder than normal for 30 minutes or more?”) (16). ST activities were assessed by a question adapted from the National Health Interview Survey (“How many days during a typical week do you perform physical activities specifically designed to strengthen your muscles such as lifting weights or doing calisthenics?”) (8). Quality of life and physical activity responses were categorized into groups approximating tertiles. Demographics, smoking status, and medical history were assessed using standard self-reported measures from CDC’s Behavioral Risk Factor Surveillance System (BRFSS) (17). Only 3 comorbidities (hypertension, diabetes, and hypercholesterolemia) were chosen to keep the instrument brief yet still assess 3 of the most common comorbidities in primary care and to compare the representativeness of our sample to the US population (18).

The survey was conducted at 2 of our general internal medicine practice sites at a single medical center in central Pennsylvania during the first 2 weeks of June 2012. Consecutive patients were handed the questionnaire during the clinic check-in process. The following script was created for front desk staff to describe the survey to patients: “For the next several weeks, we’re asking all of our patients to fill out this short questionnaire before seeing your doctor. When you’re done, please drop it in this box. Thank you very much.” No financial incentive was provided for participation, and staff did not review the questionnaires for completeness.

Bivariate associations between interest in ST and other variables were analyzed using the χ^2^ test. Logistic regression was used to describe associations between program interest and covariates, adjusting for patient demographics and medical history. Explanatory variables that were at least somewhat associated (*P* < .10) with interest in ST or those described in the literature as covariates were entered into a multiple logistic regression model. Adjusted odds ratios and 95% confidence intervals from the logistic regression models were used to describe the independent association of an explanatory variable with the outcome measure after controlling for all other potential covariates in the model. Because of a significant association between the 3 quality of life variables included in the analysis (self-reported health, number of days of poor physical health, and number of days of poor mental health), each was entered into separate logistic regression models to minimize the potential for collinearity. All analyses were performed in SPSS statistical software, version 19.0 (IBM, Inc, Armonk, New York).

The Institutional Review Board of Penn State College of Medicine determined this survey to be consistent with a quality improvement initiative, based on the Code of Federal Regulations (45 CFR part 46). It was therefore considered exempt from formal review, given that the survey was anonymous and performed with the intent of identifying programs of benefit to our patient population.

## Results

Consecutive patients (n = 570) were given a questionnaire after arriving; 483 questionnaires were returned for a response rate of 84.7%. Incomplete questionnaires (n = 69) were excluded from the multivariate analysis. Missing data varied for individual items, from less than 1% for age to 14.5% for interest in ST, resulting in 414 complete questionnaires. Overall, participants were mostly nonsmokers (90.0%) and female (64.0%) (Table 1). More than one-third (38.6%) of patients were at least 65 years of age, and most (55.1%) reported hypertension. More than one-fifth (22.2%) of patients reported no physical activity, and more than one-third (37.2%) were obese. Interest in a free ST program (55.3%) was higher than interest in a free weight control program (45.4%).

**Table 1:** Characteristics of Primary Care Patients (N = 414), Central Pennsylvania, June 2012

Variable	%
**Age, y**	
18–44	21.8
45–54	17.2
55–64	22.4
≥65	38.6
**Sex**	
Male	36.0
Female	64.0
**Smoking status**	
Smoker	10.0
Nonsmoker	90.0
**Have hypertension**	55.1
**Have diabetes**	21.0
**Have high cholesterol**	47.7
**Body mass index, kg/m^2^ (based on self-reported height and weight)^a^**	
18.5–25.0	29.8
>25.0–30.0	31.1
>30.0	37.2
**Days of aerobic activity per week**	
0	22.2
1–3	43.4
4–7	34.4
**Days of strength training per week^a^**	
0	51.3
1–3	33.2
4–7	14.5
**Self-reported health**	
Excellent, very good, or good	81.2
Fair or poor	18.8
**Days of poor physical health in past month**	
0	45.0
1–9	30.3
≥10	24.7
**Days of poor mental health in past month**	
0	50.8
1–9	27.6
≥10	21.6
**Interested in a free group strength training program**	55.3
**Interested in a free group weight loss program**	45.4

a Percentages do not total 100% because of missing data.

Most (72.8%) participants reporting more than 10 days of poor physical health in the past month were interested in the group ST program, versus 49.5% of those reporting no days of poor physical health (Table 2). Interest in ST was higher among those with more days of poor physical health, regardless of age (Figure). Approximately half (50.2%) of individuals reporting no current ST activities were interested in a ST program, compared with 64.4% of those reporting 1 to 3 days of ST per week. Interest in ST was not associated with medical history (hypertension, high cholesterol, or diabetes), smoking, days of aerobic activity, age, sex, body mass index (BMI), or self-reported health.

**Table 2:** Association Between Interest in a Free, Group-Based Strength Training Program and Primary Care Patient Characteristics (N = 414), Central Pennsylvania, June 2012

Variable	% Interested
**Age, y**	
18–44	60.6
45–54	59.5
55–64	56.1
≥65	49.7
**Sex**	
Male	49.4
Female	58.9
**Smoking status**	
Smoker	57.9
Nonsmoker	55.7
**Hypertension**	
Yes	56.8
No	53.4
**Diabetes**	
Yes	52.5
No	55.4
**High cholesterol**	
Yes	56.1
No	55.2
**Body mass index, kg/m^2^ (based on self-reported height and weight)^a^**	
18.5–25.0	53.3
>25.0–30.0	52.5
>30.0	61.3
**Days of aerobic activity per week**	
0	46.6
1–3	61.8
4–7	56.0
**Days of strength training per week**	
0	50.2^a^
1–3	64.4
4–7	57.6
**Self-reported health**	
Excellent, very good, or good	55.6
Fair or poor	54.1
**Days of poor physical health in past month**	
0	49.5^b^
1–9	54.5
≥10	72.8
**Days of poor mental health in past month**	
0	54.1
1–9	53.7
≥10	67.1

a *P* = .03, calculated using logistic regression.

b *P* = .006, calculated using logistic regression.

**Figure:**
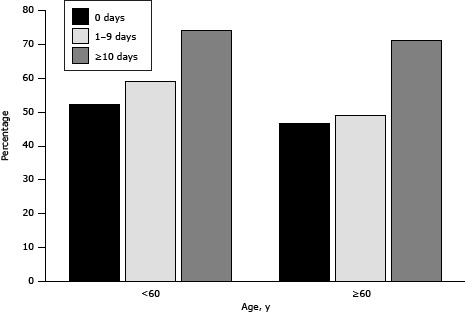
Percentage of adults interested in a free group strength training program, based on age and number of self-reported days of poor physical health in central Pennsylvania, June 2012. **Table:** 

Age, y	0 Days	1–9 Days	≥10 Days
<60	52.2	59.1	74.1
≥60	46.7	49.1	71.1

After adjusting for age, sex, smoking, hypertension, diabetes, high cholesterol, BMI, aerobic physical activity days, and ST days, the only variable that was significantly related to interest in ST was the self-reported days of poor physical health (Table 3). Participants reporting 10 or more days of poor physical health had 2.7 greater odds (95% confidence interval, 1.4–5.1) of being interested in a ST program than those reporting no days of poor physical health (*P* = .02).

**Table 3:** Association Between Interest in a Strength Training Program and Self-Reported Primary Care Patient Characteristics, Adjusted for Patient Characteristics^a^, Central Pennsylvania, June 2012

Variable	Odds Ratio (95% Confidence Interval)
**Age, y**	
18–44	1.5 (0.7–3.1)
45–54	0.9 (0.4–1.9)
55–64	0.9 (0.5–1.8)
≥65	1 [Reference]
**Sex**	
Male	1 [Reference]
Female	1.5 (0.9–2.5)
**Smoking status**	
Smoker	1 [Reference]
Nonsmoker	1.0 (0.4–2.4)
**Hypertension**	
Yes	1.0 (0.6–1.8)
No	1 [Reference]
**Diabetes**	
Yes	1.0 (0.5–1.8)
No	1 [Reference]
**High cholesterol**	
Yes	1.0 (0.6–1.8)
No	1 [Reference]
**Body mass index, kg/m^2^ (based on self-reported height and weight)^a^**	
>30.0	1.7 (0.9–3.3)
>25.0–30.0	0.9 (0.5–1.8)
18.5–2	1 [Reference]
**Days of aerobic activity per week**	
0	0.5 (0.3–1.2)
1–3	1.1 (0.6–2.1)
4–7	1 [Reference]
**Days of strength training per week**	
0	0.6 (0.3–1.5)
1–3	1.0 (0.4–2.1)
4–7	1 [Reference]
**Self-reported health**	
Excellent, very good, good	1.0 (0.6–1.9)
Fair, poor	1 [Reference]
**Days of poor physical health in last month**	
0	1 [Reference]^b^
1–9	1.2 (0.7–2.1)
≥10	2.7 (1.4–5.1)
**Days of poor mental health in past month**	
0	1 [Reference]
1–9	1.2 (0.7–2.3)
≥10	1.5 (0.7–3.0)

a Adjusted for age, sex, smoking, hypertension, diabetes, high cholesterol, body mass index, days of aerobic activity, and days of strength training.

b *P* = .02, calculated using logistic regression.

## Discussion

Most primary care patients in our sample were interested in participating in a ST program. Although no standard exists for the level of interest in a program, we were surprised that interest in ST was higher than interest in a weight control program. This finding may be because many adults have tried to lose weight and realize that it is very difficult, yet fewer have tried ST. Andreyeva and colleagues observed that 44.1% of men and 64.9% of women respondents to the BRFSS were trying to lose weight at the time of the survey, suggesting that many adults have experience with trying to lose weight (19). We believe that the high level of interest in ST in this study is because most of our patients were aged 55 years or older. Older adults are aware that certain activities are harder for them (eg, walking, lifting, pushing) and may believe that ST can help. Schoenborn and Heyman observed, for example, that 25.0% of adults over 55 reported trouble walking one-quarter mile and that difficulty walking rises sharply with age (20). Despite a lack of understanding of why interest in ST is high, these results give us confidence that this interest may lead to high rates of participation, given the great number of people who pursue weight loss programs (eg, Weight Watchers) each year.

People who reported 10 or more days of poor physical health had 2.7 greater odds of being interested in a ST program compared with those reporting no days of poor health. Studies show that older and less healthy adults are less likely to do ST, so we expected that older adults and less healthy adults would also be less interested. Ciccolo and colleagues, for example, observed that people with better self-reported health were 2.32 times as likely to meet recommended levels of ST compared with those with worse self-reported health (21). The findings in our study suggest that, although older and less-well adults may do less ST, their interest in ST may actually be greater. More than 8 times as many older adults (27%) fear the loss of their independence than fear their own death (3%) (22), so our findings indicate that older adults with poorer physical health recognize that certain activities are harder for them to do and believe that ST may help. This observation also suggests that primary care practices could screen patients using the days of poor physical health measure to identify and refer patients who have the highest interest in, and possibly the most to gain, from ST programs.

Despite a high response rate and a timely question aligned with the growing focus on population health in the Affordable Care Act, this study has many limitations. First, it was performed in only 1 location in central Pennsylvania, so the results may not be generalizable to other settings. Second, it is not clear whether the high rates of interest expressed by patients would translate into high rates of future participation. A review of 23 worksite studies observed a mean participation rate of 33% for health promotion interventions (eg, diet, exercise, smoking cessation) (23), suggesting that if programs are offered, many individuals participate. Future studies could address this concern by asking survey participants whether they were interested in participating “now” and, if so, asking for contact information. Although we suspect this shorter-term participation rate would be lower, knowing the ratio between interest, as determined in this study, and shorter-term participation may help with program planning. Third, in an effort to keep response rates high, we measured few covariates. This increases the potential for confounding from unmeasured variables (eg, income, ethnicity, marital status). We purposefully did not ask household income, as this item has the highest nonresponse rate (10%–20%) of any demographic question (24).

Despite these limitations, our findings suggest that interest in free ST programs is high and greater among those with poorer physical health. ST programs have high effect sizes and high rates of adherence, making them ideal for improving public health (25). Our study suggests that primary care patients are interested in participating in such programs and those who need them the most are also the most interested.

On the basis of these findings, primary care practices may consider ways to expand access and referrals to ST programs in their communities. Because most seniors do not have insurance (eg, Medicare supplements) that grants them access to free programs (eg, Silver Sneakers), our practices created our own evidence-based program (7) that could be delivered inexpensively to a large patient population. Our program, Band Together (www.BTPennState.org), uses trained volunteers (26) to lead a ST program that uses inexpensive resistance bands and is held in donated spaces (eg, churches, senior housing, senior centers). Our practices also screen for poor physical health and specifically encourage those with more days of poor physical health to consider participating in a ST program. By referring patients who are both most interested and in greatest need, ST programs can effectively improve the public’s health.
